# Genetic association between TNF-α G-308A and osteoarthritis in Asians: A case–control study and meta-analysis

**DOI:** 10.1371/journal.pone.0259561

**Published:** 2021-11-04

**Authors:** Chih-Chien Wang, Chih-Yun Huang, Meng-Chang Lee, Dung-Jang Tsai, Chia-Chun Wu, Sui-Lung Su

**Affiliations:** 1 Department of Orthopedics, Tri-Service General Hospital and National Defense Medical Center, Taipei, Taiwan; 2 School of Public Health, National Defense Medical Center, Taipei, Taiwan; 3 Artificial Intelligence of Things Center, Tri-Service General Hospital, National Defense Medical Center, Taipei, Taiwan, ROC; Shanghai Jiao Tong University, CHINA

## Abstract

**Background:**

Osteoarthritis (OA) is an important health issue in elderly people. Many studies have suggested that genetic factors are important risk factors for OA, of which tumor necrosis factor-α (TNF-α) is one of the most examined genes. Moreover, several studies have investigated the relationship between TNF-α G-308A polymorphisms and OA risk, but consistent results have not been obtained.

**Objective:**

This study examines the association between TNF-α G-308A polymorphisms and knee OA. Moreover, meta-analysis and trial sequential analysis (TSA) was used to determine whether this is a susceptibility gene for knee OA.

**Methods:**

Between 2015 and 2019, 591 knee OA cases and 536 healthy controls were recruited. The Kellgren–Lawrence grading system was used to identify the knee OA cases. A meta-analysis was conducted including related studies published until 2020 from PubMed, Embase, and previous meta-analysis to improve the evidence level of the current study. The results were expressed as odds ratios (ORs) with corresponding 95% confidence intervals (CI) to evaluate the effect of this polymorphism on knee OA risk. The TSA was used to estimate the sample sizes required in this issue.

**Results:**

A nonsignificant association was found between the AA genotype and knee OA [adjusted OR, 0.84; 95% CI, 0.62–1.15) in the recessive model] in the present case–control study, and analysis of other genetic models showed a similar trend. After adding the critical case–control samples for Asians, the TNF-α G-308A, AA genotype exhibited 2.57 times more risk of developing arthritis when compared with the GG + GA genotype (95% CI, 1.56–4.23), and the cumulative samples for TSA (*n* = 2182) were sufficient to obtain a definite conclusion.

**Conclusions:**

The results of this meta-analysis revealed that the TNF-α G-308A, AA genotype is a susceptible genotype for OA in the Asian population. This study integrated all current evidence to arrive at this conclusion, suggesting that future studies on Asians are not required.

## 1. Introduction

Osteoarthritis (OA) is the most common joint disease that is the main cause of disability among elderly people globally [1]. Genetic factors are particularly important, and the heritability of knee OA is around 45% [2]. Moreover, a previous study suggested that OA is primarily influenced by genetic risk factors due to common population polymorphisms in multiple genes [3]. Thus, identifying more candidate genes and evaluating their effects are desirable.

Tumor necrosis factor-α (TNF-α) is located in the major histocompatibility complex III region (6p21.3) on chromosome 6 [4]. TNF-α participates in inflammatory responses and induces similar responses in other cells [5]. Its signal is mainly transmitted through TNF receptor-1 [6, 7], thereby regulating other cytokines and growth factors to elicit their effects on the body [8]. The increased inflammation or decreased anabolism results in cartilage structure destruction. Therefore, TNF-α is a susceptibility OA marker [9]. Commonly discussed TNF-α single nucleotide polymorphisms (SNPs) include G-308A and G-238A. Previous studies found that the expression level of TNF-α G-308A gene polymorphism has the highest correlation with OA [10] and that an increased OA risk prevails in the recessive genetic model, which only exists among the Asian population [11]. Therefore, this study aimed to examine the relationship between TNF-α G-308A gene polymorphism and OA.

Twelve studies have currently investigated the association between G-308A polymorphism and knee OA [10, 12–22]. However, no satisfactory consensus has been reached. Four studies considered that the minor allele (A allele) carriers had a higher risk of knee OA [10, 14, 21, 22]. One study considered that the minor allele (A allele) carriers had a lower risk of knee OA [17]. Seven studies also showed a null association between TNF-α G-308A polymorphism and knee OA [12, 13, 15, 16, 18–20]. The trial sequential analysis (TSA) provided an opportunity to evaluate whether the most recent conclusions are supported by the current cumulative samples [23]. The sample size provided by the currently available studies is only 1,533, 1,647, and 600 in Asians, Caucasians, and Egyptians [10, 12–22], which is not enough for obtaining a decisive conclusion. Thus, this study aims to conduct a case–control study to validate the association between TNF-α G-308A polymorphism and knee OA in Taiwan. A meta-analysis was also performed to improve the evidence level and evaluate whether the latest conclusions are supported by the current cumulative samples using TSA.

## 2. Materials and methods

### 2.1 Case–control study

#### 2.1.1 Ethical issues

This study was approved by the Institutional Review Board (TSGH-2-102-05-028) of the Tri-Service General Hospital (TSGH). Volunteers signed the consent form after the investigators explained the study.

#### 2.1.2 Subjects

This study enrolled 1,127 participants (591 cases and 536 controls) comprising individuals ≥65 years old. All received Taipei City senior medical check-ups between January 2015 and December 2019 at the TSGH, a medical teaching hospital at the National Defense Medical Center, Taipei, Taiwan. The check-up is a government-driven welfare program for individuals ≥65 years old who are registered Taipei City residents for >1 year.

Patient information was examined while the participants underwent check-ups. All participants who received the study information, understood the process, and provided written consent were enrolled. Participants were excluded if sufficient blood samples were drawn. The exclusion criteria were patients who had no knee X-ray or genotype data.

#### 2.1.3 Genomic DNA extraction and genotyping

Approximately 10 mL of peripheral blood was intravenously extracted from participants by a physician or nurse. Genomic DNA was isolated using standard procedures with proteinase K (Invitrogen, Carlsbad, CA, USA) digestion and phenol/chloroform methods. The rs1800629 SNP was genotyped using the iPLEX Gold SNP genotyping method. Genotyping was performed under blind conditions. At least 10% of the samples were randomly selected for repeated genotyping to validate the results, and the concordance rate was 99%.

#### 2.1.4 Statistical analysis

Continuous variables of the general demographic data were expressed as mean and standard deviation using Student’s *t*-test. Differences in genotype and allele frequencies between knee OA patients and healthy controls were tested using the χ^2^ test. Odds ratios (ORs) and 95% confidence intervals (CIs) for the risk of knee OA were calculated using logistic regression. The primary analysis was the association test based on the recessive model assumption [11]. The other assumptions of allele type, genotype, and dominant models were also presented. A *p* value of <0.05 was considered significant. R 3.4.4 was used for statistical analyses.

### 2.2 Meta-analysis

#### 2.2.1 Search methods and criteria for study consideration

The PRISMA checklist and Meta-analysis on Genetic Association Studies Checklist are described in S1 Table [24]. The samples examined in this study comprised the general population, and the correlation between TNF-α G-308A and OA risk was examined. This study used synonyms of “TNF α G-308A” and “osteoarthritis” to search PubMed, EMBASE, and Cochrane databases for papers until 17 November 2020 (see S2 Table for details). The language of the articles was limited to English. In addition, the papers included in the meta-analysis studies were manually examined to avoid the omission of important papers. The papers conforming to the following conditions were included in the current study: (1) case–control studies or cross-sectional studies; (2) clear diagnostic standard for the case group, for e.g., case group (KL ≥ 2) and control group (KL < 2); and (3) samples were from adults ≥18 years old.

#### 2.2.2 Data extraction

Two reviewers (Chih-Yun Huang and Sui-Lung Su) were responsible for the independent extraction of literature data in this study. The data extracted included the last name of the first author, publication year, country, ethnicity of the study population, and gene distribution in the case and control groups. All extracted papers were assessed using the Newcastle–Ottawa Scale, and the details are shown in S1 Table.

#### 2.2.3 Statistical analysis

All included papers were described using appropriate proportions or mean values. The current meta-analysis used ORs with 95% CIs to examine the correlation between TNF-α G-308A and OA. The *I*^2^ test was used to assess heterogeneity. *I*^2^ >50% denoted moderate to high heterogeneity. Egger’s regression and a funnel plot were used to examine the symmetry after combination. The primary analysis was the association test based on the recessive model assumption [11]. The other assumptions of allele type and dominant models were also presented. The random-effects model was used to combine the results. The significance level of this study was set as 0.05. The “metafor” [25] and “meta” [26] packages of R software version 3.3.1 were used. In addition, sensitivity analysis was used to exclude papers that did not conform to the Hardy–Weinberg equilibrium or had irrational data. Moreover, the current study employed TSA to validate whether the meta-analysis result was a definite conclusion [27]. TSA was used for stratification analysis based on race (Caucasian, Asian, and Egyptian). Type 1 error, power, and heterogeneity were set at 0.05, 0.8, and 0%, 65%, and 0% in Caucasian, Asian, and Egyptian populations, respectively. A review of the previous literature showed that the OR of correlation between TNF-α G-308A and OA was around 1.5. In this study, OR was set as 1.5 because the A allele is a possible risk factor. The Taiwan Biobank database and 1000 Genome database were used as references for minor allele frequency, which was 0.11, 0.069, and 0.12 for Asians, Caucasians, and Egyptians, respectively.

## 3. Results

### 3.1 Case–control study

Table 1 shows the distribution of general demographic variables of the case–control population. This enrolled 1,127 subjects, including 536 subjects in the control group with a mean age of 71.78 ± 6.85 years old (264 men and 272 women) and 591 subjects in the case group with a mean age of 73.57 ± 7.36 years old (214 men and 377 women). The proportion of males was lower in the case group than in the control group (*p* < 0.001), and the age and body mass index of the case group were higher than those of the control group (*p* < 0.001 and *p* = 0.019). Table 2 shows the correlation between TNF-α G-308A and knee OA in the case and control groups. The distribution of the A allele did not demonstrate any significant differences between the control and case groups (*p* = 0.091) and did not exhibit a significant correlation with OA (OR, 2.74; 95% CI, 0.85–8.81). The dominant and recessive models were further used to validate the results and similarly did not find any significant differences after correcting for covariates. Therefore, the current case–control study found that TNF-α G-308A does not have a significant correlation with OA. The samples from the case–control study were included in the meta-analysis and further employed TSA validation to see if definite conclusions could be obtained for Asian samples to increase the power of evidence for the meta-analysis.

**Table 1:** Distribution of basic demographic data of the study subjects.

	Knee osteoarthritis group (*n* = 591)	Control group (*n* = 536)	*p* value
Sex			<0.001*
Male	214 (36.2%)	264 (49.3%)	
Female	377 (33.8%)	272 (50.7%)	
Age (mean ± SD)	73.57 ± 7.36	71.78 ± 6.85	<0.001*
BMI	24.62 ± 3.62	24.12 ± 3.32	0.019*
SBP (mmHg)	132.48 ± 17.71	131.56 ± 17.48	0.436
DBP (mmHg)	77.37 ± 12.37	76.59 ± 10.88	0.292
KL grade (%)			<0.001*
KL = 0		22 (4.1%)	
KL = 1		514 (95.9%)	
KL = 2	437 (73.9%)		
KL = 3	84 (14.2%)		
KL = 4	70 (11.8%)		

Knee osteoarthritis group, KL ≥ 2; control group; KL < 2.

*BMI* body mass index, *SBP* systolic blood pressure, *DBP* diastolic blood pressure.

*p value < 0.05.

**Table 2:** Correlation between TNF-α G-308A gene polymorphism and knee osteoarthritis risk.

	Knee osteoarthritis group (*n* = 591)	Control group (*n* = 536)	Crude OR 95% CI	*p* value	Adjusted OR^a^ 95% CI	*p* value
**Genotype**				0.134		0.231
GG	484 (81.9%)	428 (79.9%)	1		1	
GA	95 (16.1%)	104 (19.4%)	0.81 (0.59–1.10)	0.173	0.78 (0.56–1.07)	0.121
AA	12 (2%)	4 (0.7%)	2.65 (0.85–8.29)	0.093	2.61 (0.81–8.41)	0.109
**Allele model**						
G	1063 (89.7%)	960 (89.6%)	1		1	
A	119 (10.3%)	112 (10.4%)	2.76 (0.88–8.60)	0.081	2.74 (0.85–8.81)	0.091
**Dominant model**						
GG	484 (81.9%)	428 (79.9%)	1		1	
GA + AA	107 (18.1%)	108 (20.1%)	0.98 (0.75–1.29)	0.906	0.84 (0.62–1.15)	0.280
**Recessive model**						
GG + GA	579 (98%)	532 (99.3%)	1		1	
AA	12 (2%)	4 (0.7%)	0.88 (0.65–1.18)	0.383	0.84 (0.62–1.15)	0.280

Knee osteoarthritis group, KL ≥ 2; control group, KL < 2.

*OR* odds ratio.

^a^Adjusted by gender, age, and body mass index.

*p value < 0.05.

### 3.2 Meta-analysis

**Fig 1:**
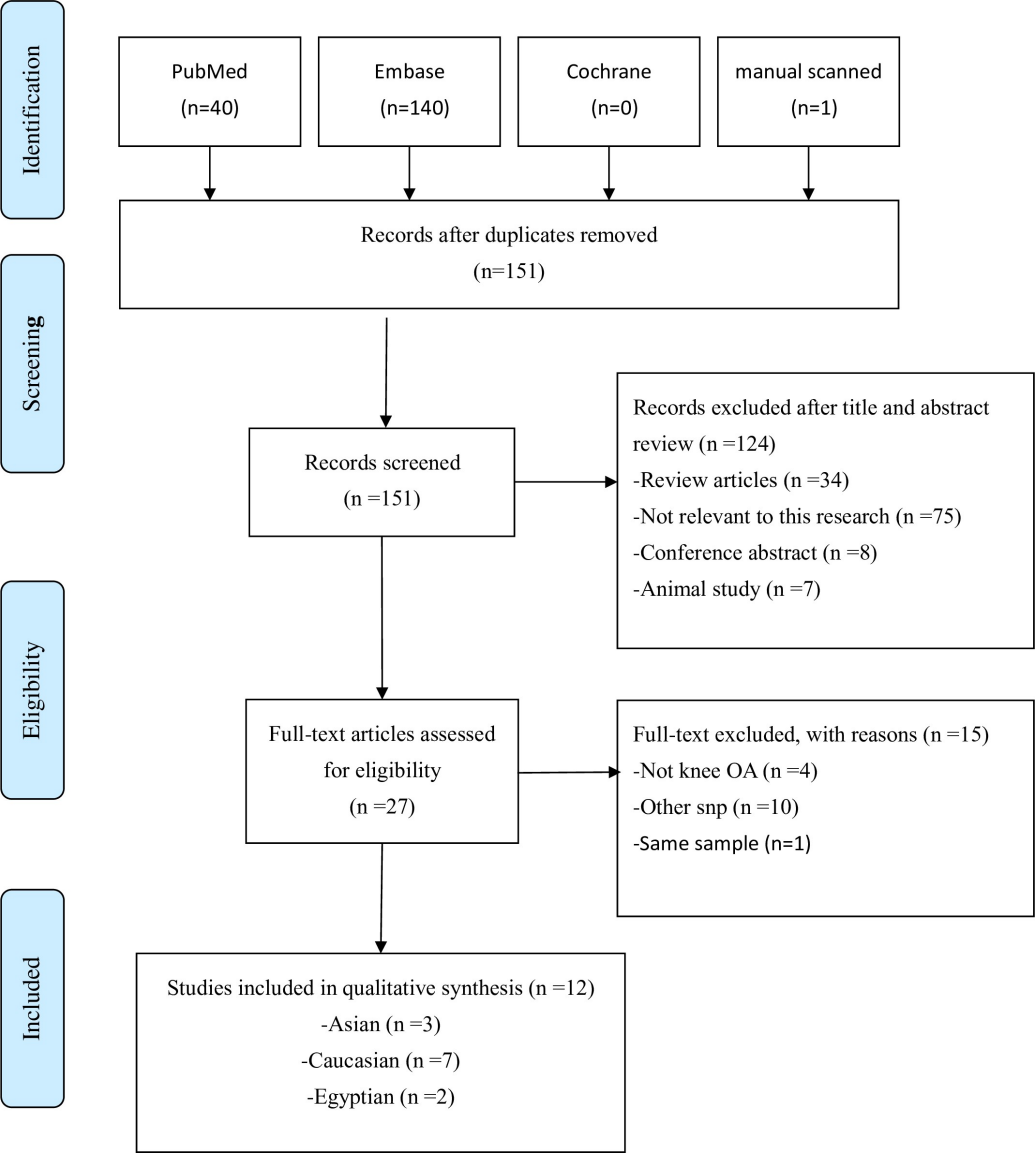
Flow diagram of the identification process for eligible studies.

Fig 1 presents the search flowchart of this study. Forty papers were first searched from PubMed. Another 141 papers from Embase were then obtained, and manual searches from other meta-analyses were done in 151 papers after excluding repeated papers. Screening based on the title and abstract was conducted, resulting in the exclusion of 34 retrospective or meta-analysis papers, 75 papers unrelated to this study, eight conference abstracts, and seven animal studies. Based on the screening of the entire manuscripts, four non-knee OA papers, 10 papers containing gene polymorphisms that were not defined in this study, and one paper with repeated samples were noted. Finally, 12 papers were included in the analysis. S3–S5 Tables show the general description of the papers included in the meta-analysis, ethnicities studied in the papers, and the paper quality evaluation.

**Fig 2:**
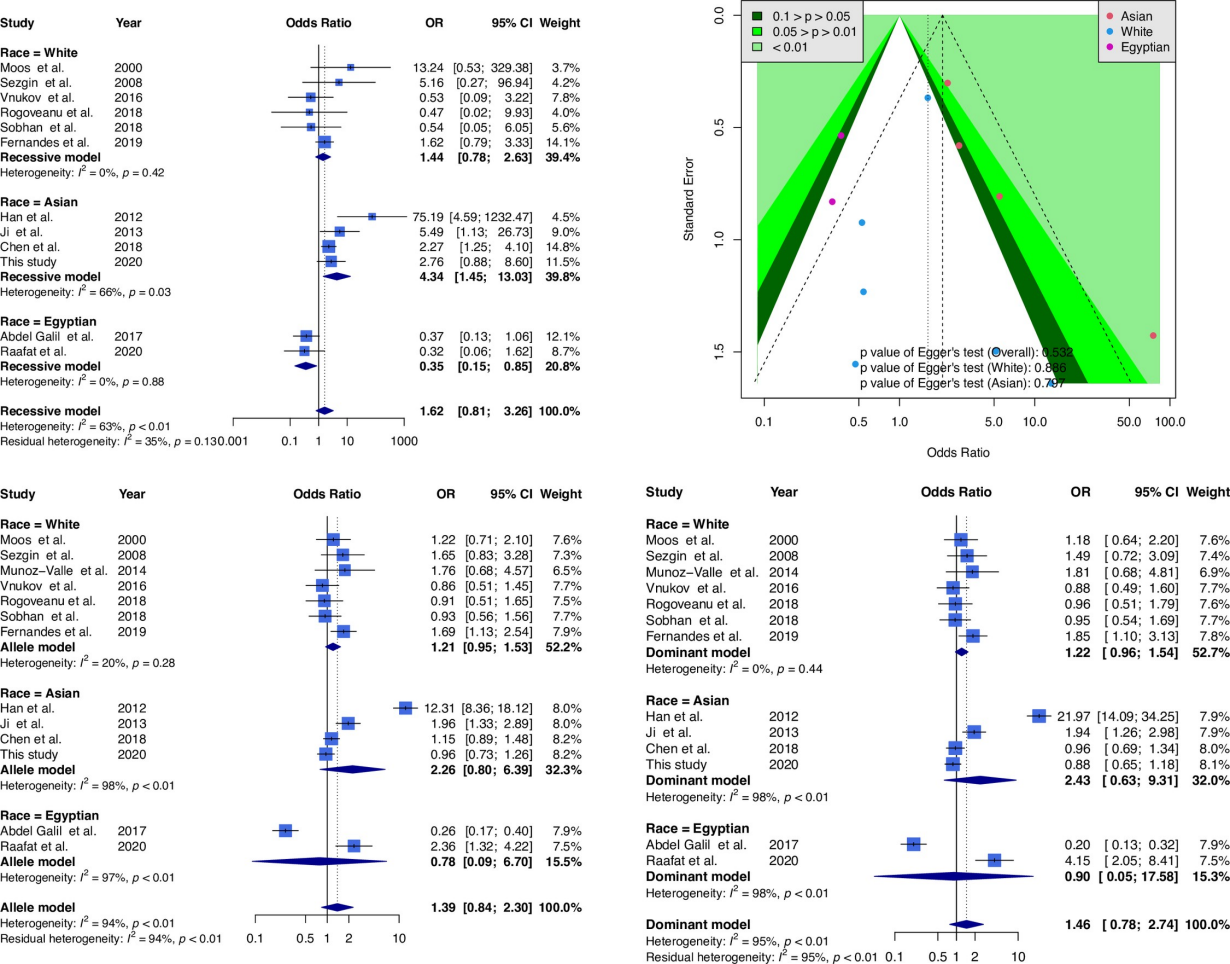
Forest plot and funnel plot of the correlation between TNF-α G-308A and osteoarthritis. Selected results from the meta-analysis of TNF-α G-308A and knee osteoarthritis. The *top left subplot* is a forest plot based on a recessive model assumption (AA vs. GG + AG), and the *top right subplot* is a funnel plot based on the recessive model assumption. The results obtained with the allele (reference, G allele) and dominant (AG + AA vs. GG) models are presented at the *bottom*.

Fig 2 shows the meta-analysis results. The results of combining 12 samples for the recessive model (GG + GA genotype vs. AA genotype) did not reach significance (OR = 1.62; 95% CI, 0.81–3.26). Based on ethnic stratification, four Asian samples with significant results (OR = 4.34; 95% CI, 1.45–13.03), seven Caucasian samples and with nonsignificant results (OR = 1.44; 95% CI, 0.78–2.63), and two Egyptian samples with significant results (OR = 0.35; 95% CI, 0.15–0.85) were noted. A funnel plot was used to demonstrate the association between ORs and standard error in the recessive model, with each point representing a study. No significant asymmetry was discovered between the articles. The other analyses based on allele and dominant model revealed results similar to those of the recessive model.

### 3.3 TSA sample estimation

**Fig 3:**
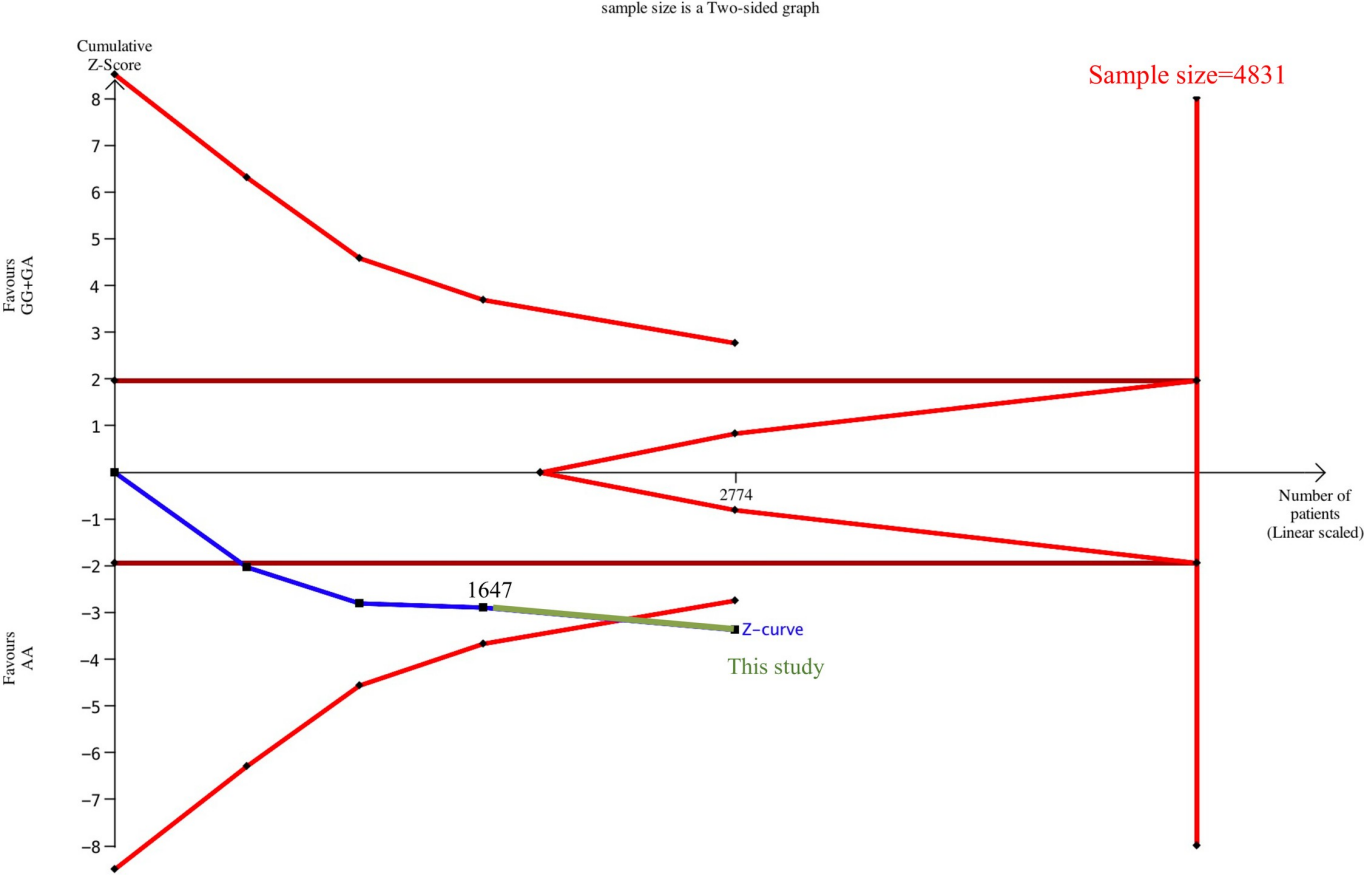
Estimation of the Asian sample size for correlation between TNF-α G-308A and osteoarthritis. A trial sequential analysis using a dominant model assumption was performed. Detailed settings: significance level = 0.05, power = 0.8, least extreme odds ratio to be detected = 1.5, minor allele frequency = 0.11, and *I*^2^ (heterogeneity) = 65%.

The sample size for Caucasians was 1,533 patients after TSA estimation without significant correlation between TNF-α G-308A and OA, and the cumulative sample size did not reach the target sample size (2,528). Therefore, definite results could not be obtained in the meta-analysis of Caucasians (S1 Fig). The cumulative sample size for Asians was 1,647 patients before adding samples from this study, and a significant correlation was noted between TNF-α G-308A and OA. The cumulative sample size for Asians was 2,774 after the inclusion of this study, but the cumulative sample size did not reach the target sample size (4,831). Therefore, definite results could not be obtained in the meta-analysis of Asians (Fig 3). The cumulative sample size for Egyptians was 600 patients, and TNF-α G-308A showed a significant correlation with OA. However, the cumulative sample size did not reach the target sample size (1,617). Therefore, definite results could not be obtained in the meta-analysis of Egyptians (S2 Fig).

### 3.4 Sensitivity analysis

**Fig 4:**
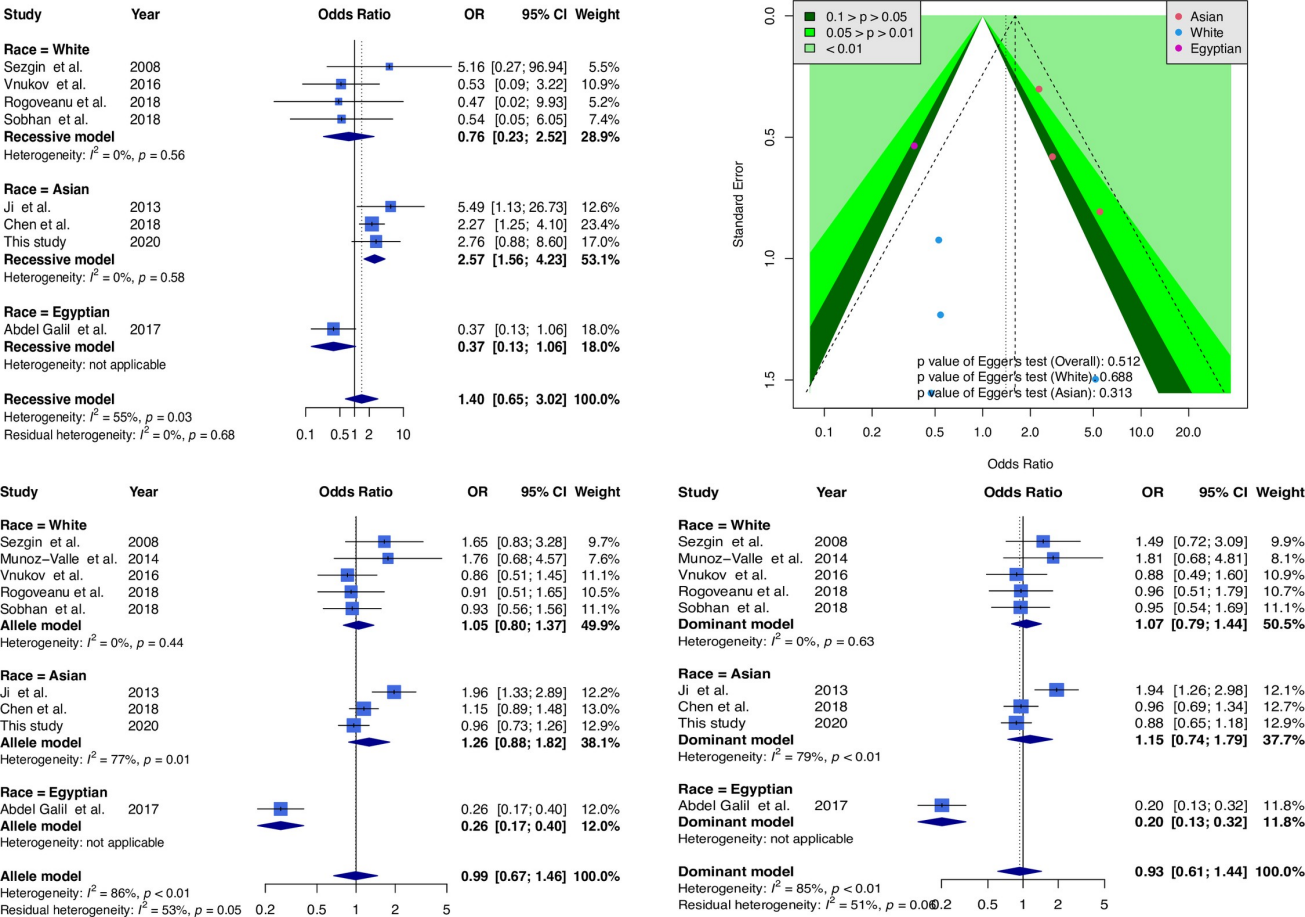
Forest plot and funnel plot for sensitivity analysis of the correlation between TNF-α G-308A and osteoarthritis. Selected results from the meta-analysis of TNF-α G-308A and knee osteoarthritis. The *top left subplot* is a forest plot based on a recessive model assumption (AA vs. GG + AG), and the *top right subplot* is a funnel plot based on the recessive model assumption. The results obtained with the allele (reference. G allele) and dominant (AG + AA vs. GG) models are presented at the *bottom*.

The study by Han et al. showed an OR ratio that was too high [14]. However, the studies by Moos et al., Fernandes et al., and Raafat et al. did not conform to the Hardy–Weinberg equilibrium [12, 21, 22], which may affect the meta-analysis results; thus, these papers were excluded. The funnel plot, forest plot, and TSA results graph after excluding these papers were as follows: eight samples were combined in the recessive model (GG + GA genotype vs. AA genotype) after the exclusion, and the model did not reach significance (OR = 1.40; 95% CI, 0.65–3.02). Three Asian samples with significant results (OR = 2.57; 95% CI, 1.56–4.23) and four Caucasian samples with nonsignificant results (OR = 0.76; 95% CI, 0.23–2.52) were noted based on ethnic stratification. In addition, no hidden publication bias in the papers exists, and Fig 4 shows the forest and funnel plots.

**Fig 5:**
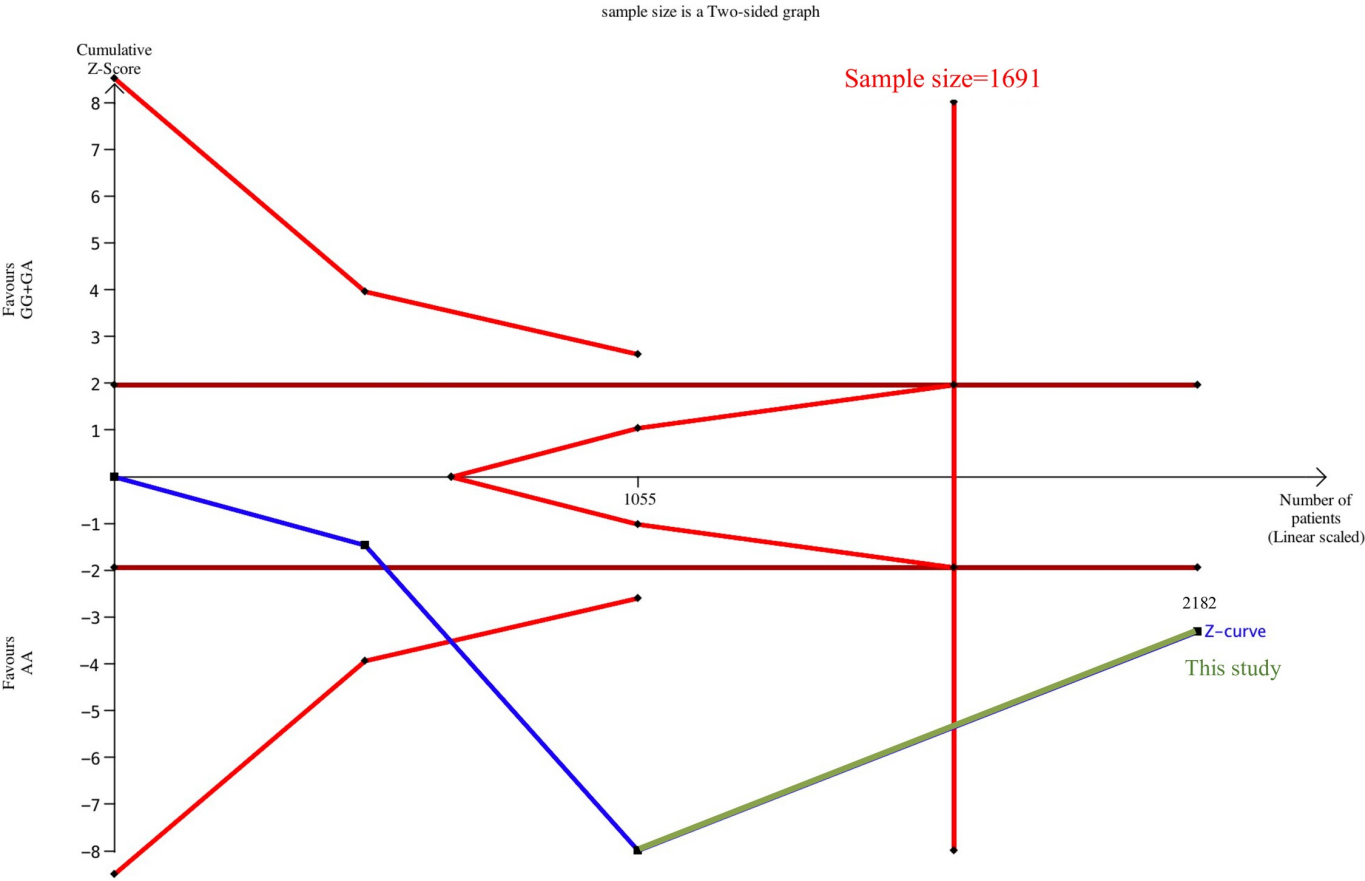
Estimation of Asian sample size for sensitivity analysis of the correlation between TNF-α G-308A and osteoarthritis. A trial sequential analysis using a dominant model assumption was performed. Detailed settings: significance level = 0.05, power = 0.8, least extreme odds ratio to be detected = 1.5, minor allele frequency = 0.11, and *I*^2^ (heterogeneity) = 0%.

TSA estimation was performed after exclusion. Consequently, 981 cumulative Caucasian samples were noted without significant correlation between TNF-α G-308A and OA, and the sample size was insufficient to obtain a definite conclusion (S3 Fig). The cumulative sample size for Asians was 1,055 patients before adding samples from this study, and a significant correlation was noted between TNF-α G-308A and OA. The cumulative sample size for Asians was 2,182 after the inclusion of this study, and the cumulative sample size reached the target sample size (1,691; Fig 5). This result signified that TNF-α G-308A and OA were also significantly correlated in Asians, and a decisive conclusion could be confirmed. Thus, the case–control samples in this study provided critical evidence for establishing a decisive conclusion.

## 4. Discussion

The current case–control study identified no significant correlation between TNF-α G-308A and OA risk. This result is consistent with some of the previous studies [12, 13, 15, 16, 18–20]. However, other studies have indicated that TNF-α G-308A is correlated with OA risk [10, 14, 17, 21, 22]. G-308A was changed from guanine (G) to adenine (A) [28], and the performance of TNF-α is affected when the G allele mutates into the A allele [14, 29]. TNF-α enhances articular chondrocyte stiffness and impaired contraction function and is associated with OA [30]. Increased TNF-α mRNA expression in cartilage increases inflammation, thereby stimulating matrix metalloproteinase in chondrocytes, degrading extracellular matrix in cartilage, and disrupting cartilage structure integrity [31, 32]. Recent studies have shown that TNF-α is the most important mediator that alters the cartilage matrix degradation and balance in patients with arthritis and ultimately leads to cartilage degradation [33]. Thus, the number and sample size of the selected studies are suggested to be small and insufficient to obtain a conclusive result. Additionally, N4-acetylcytidine (ac4C) modification of mRNA enhances mRNA stability and translation efficiency which correlate with the occurrence, development, and prognosis of diseases. Escalating ac4C levels in the urine may also be associated with inflammatory responses [34]. More investigations on TNF-α and ac4C would help clarify the role of TNF-α in the mechanisms of OA.

The current meta-analysis found an increased risk by using the recessive model analysis. However, a subgroup analysis found that the relationship between TNF-α-308 polymorphism and OA risk only existed among Asians and not among Caucasians, which is consistent with the results of previous meta-analyses [11, 18, 19]. Another study also found different results in different ethnic populations. The study group of North and South Indians separately explored the relationship between TNF-α G-308A and diseases. The results showed an association between southern Indians’ genotypes and diseases, but no association was found in northern Indians, confirming that TNF-α G-308A gene variants are affected by race [35]. Heterogeneity was significantly decreased and the TNF-α G-308A polymorphism was associated with OA risk in the Asian population when the analysis stratified by ethnicity was performed. This finding may be attributed to the AA genotype that is more important for knee OA susceptibility in Asians than in Caucasians. The TSA result showed that the current cumulative samples were sufficient to reach a decisive conclusion in Asians but not in Caucasians. This is believed to be the first meta-analysis study to explore the correlation between TNF-α G-308A and knee OA using TSA.

In the precision medicine era, genomic and environmental factors that are significantly related to disease phenotypes may be used as biomarkers for risk prediction and early disease diagnosis [36–46]. Well-trained deep learning models based on these biomarkers have been widely used as tools to identify diseases [47]. The next goal of the current study is to use deep learning approaches to predict OA or its progression upon genomic variants.

The current study has three major strengths: (1) none of the previous meta-analyses estimated whether the cumulative sample size is sufficient to obtain a definite conclusion. This study employed TSA estimation and found that definite conclusions can be obtained based on the Asian sample size. (2) This study used the random-effects model to combine results and avoid serious errors caused by model selection based on heterogeneity [48]. (3) In this study, two evaluators used the Newcastle–Ottawa Scale to evaluate the paper quality and excluded those that did not conform to Hardy–Weinberg equilibrium or had irrational data, thereby enhancing the reliability of the results. However, this study has some limitations. (1) The current study only included papers published in English, and those in other languages were not included in the meta-analysis. This omission may result in bias in the combined results. (2) The high heterogeneity could not be explained, which may imply potential gene–gene and gene–environment interactions. Further research is required to shed light on complete population characteristics for future meta-analysis. Moreover, the issues (e.g., publication bias from smaller-scale research or heterogeneity) can be assessed or processed by utilizing quality assessment scores [49–53] or stratification [54–56]. (3) This study shows limited causality to explore the genetic variant effect on OA development. In the future, linkage disequilibrium score and Mendelian randomization should be employed to see if the TNF-α G-308A genetic polymorphism or other SNPs in this gene may causally trigger the development of OA through mediating the expression of this gene in cartilage [57–61].

## 5. Conclusion

In conclusion, the definite null relationship between TNF-α G-308A and knee OA was validated in this case–control study. However, this meta-analysis demonstrated that the AA genotype of the TNF-α G-308A polymorphism increased the risk of OA in Asians. A decisive conclusion could be obtained, for which the current case–control samples provided critical evidence. In contrast, TNF-α G-308A polymorphism was not associated with knee OA risk in Caucasians. Further studies with a large sample size are necessary to validate whether TNF-α gene polymorphism contributes to knee OA susceptibility in Caucasians.

## Supporting information

S1 Table PRISMA 2009 checklist. (DOC)

S2 Table Search strategies and detailed records. (DOCX)

S3 Table General description of papers included in meta-analysis. (DOCX)

S4 Table Extracted information from papers included in the meta-analysis. (DOCX)

S5 Table TNF-α G-308A paper quality evaluation. (DOCX)

S1 Fig Estimation of Caucasian sample size for correlation between TNF-α G-308A and osteoarthritis. A trial sequential analysis was performed using a dominant model assumption. Detailed settings: significance level = 0.05, power = 0.8, least extreme odds ratio to be detected = 1.5, minor allele frequency = 0.069, and *I*^2^ (heterogeneity) = 0%.
(TIF)

S2 Fig Estimation of Egyptian sample size for correlation between TNF-α G-308A and osteoarthritis. A trial sequential analysis was performed using a dominant model assumption. Detailed settings: significance level = 0.05, power = 0.8, least extreme odds ratio to be detected = 1.5, minor allele frequency = 0.12, and *I*^2^ (heterogeneity) = 0%.
(TIF)

S3 Fig Estimation of Caucasian sample size for sensitivity analysis of the correlation between TNF-α G-308A and osteoarthritis. A trial sequential analysis was performed using a dominant model assumption. Detailed settings: significance level = 0.05. power = 0.8, least extreme odds ratio to be detected = 1.5, minor allele frequency = 0.069, and *I*^2^ (heterogeneity) = 0%.
(TIF)
