# Supplementary material for: Genetic association between TNF-α G-308A and osteoarthritis in Asians: A case–control study and meta-analysis
Source: PLoS One. 2021 Nov 4;16(11):e0259561. doi: 10.1371/journal.pone.0259561 (PMC8568107; doi:10.1371/journal.pone.0259561)
Supplement: S2 Table — (DOCX) [file pone.0259561.s002.docx]

**S2 Table. Search strategies and detailed records.**

| **Relevant text of TNF-α G-308A**   1. tumor necrosis factor-alpha 2. tnf-α 3. TNF-α 4. cachectin 5. cachectin-tumor necrosis factor 6. tumor necrosis factor ligand superfamily member 2 7. tumor necrosis factor 8. tnf superfamily, member 2 9. tnfalpha 10. tnf-alpha 11. Polymorphisms, Genetic 12. Genetic Polymorphisms 13. Genetic Polymorphism 14. polymorphism 15. polymorphisms 16. nucleotide polymorphism, single 17. nucleotide polymorphisms, single 18. polymorphisms, single nucleotide 19. single nucleotide polymorphisms 20. SNPs 21. Single Nucleotide Polymorphism | 1. rs1800629 2. G-308A 3. (1 or 2 or 3 or 4 or 5 or 6 or 7 or 8 or 9 or 10) and (11 or 12 or 13 or 14 or 15 or 16 or 17 or 18 or 19 or 20 or 21) or (22) or (23)   **Relevant text of Osteoarthritis**   1. osteoarthritis 2. osteoarthritides 3. osteoarthrosis 4. osteoarthroses 5. arthritis, degenerative 6. arthritides, degenerative 7. degenerative arthritides 8. degenerative arthritis 9. arthrosis 10. arthroses 11. osteoarthrosis deformans 12. 25 or 26 or 27 or 28 or 29 or 30 or 31 or 32 or 33 or 34 or 35   **Combined (Final Strategy)**   1. 24 and 36 |
| --- | --- |

MeSH Browser: <http://www.nlm.nih.gov/mesh/MBrowser.html>

PubMed: <http://www.ncbi.nlm.nih.gov/pubmed>

Cochrane Library: <http://www.thecochranelibrary.com>

Embase: https://www.embase.com
