# Supplementary material for: Genetic association between TNF-α G-308A and osteoarthritis in Asians: A case–control study and meta-analysis
Source: PLoS One. 2021 Nov 4;16(11):e0259561. doi: 10.1371/journal.pone.0259561 (PMC8568107; doi:10.1371/journal.pone.0259561)
Supplement: S3 Table — (DOCX) [file pone.0259561.s003.docx]

**S3 Table.** 統合分析納入文獻之基本描述

| Author | Year | Country | Ethnicity | Study design | **Definition of OA** |
| --- | --- | --- | --- | --- | --- |
| **Moos** | **2000** | Germany | Caucasian | **Case-control study** | **KL ≥ 2** |
| **Sezgin** | **2008** | Turkey | Caucasian | **Case-control study** | **KL ≥ 2** |
| Han | **2012** | Korea | Asian | **Case-control study** | **KL ≥ 2** |
| Ji | **2013** | China | Asian | **Case-control study** | **KL ≥ 2** |
| Munoz-Valle | **2014** | Mexico | Caucasian | **Case-control study** | **KL ≥ 2** |
| Vunkov | **2016** | Russia | Caucasian | **Case-control study** | **KL ≥ 2** |
| Abdel Galil | **2017** | Egypt. | Egyptian | **Case-control study** | **KL ≥ 2** |
| Rogoveanu | **2018** | Romania. | Caucasian | **Case-control study** | **KL ≥ 2** |
| Sobhan | **2018** | Iran | Caucasian | **Case-control study** | **KL ≥ 2** |
| Chen | **2018** | China | Asian | **Case-control study** | **KL ≥ 2** |
| Fernandes | **2019** | Brazil | Caucasian | **Case-control study** | **KL ≥ 2** |
| Raafat | **2020** | Egypt | Egyptian | **Case-control study** | **KL ≥ 2** |
| This study | **2020** | Taiwan | Asian | **Case-control study** | **KL ≥ 2** |
