# Supplementary material for: Genetic association between TNF-α G-308A and osteoarthritis in Asians: A case–control study and meta-analysis
Source: PLoS One. 2021 Nov 4;16(11):e0259561. doi: 10.1371/journal.pone.0259561 (PMC8568107; doi:10.1371/journal.pone.0259561)
Supplement: S4 Table — (DOCX) [file pone.0259561.s004.docx]

**S4 Table.** 統合分析納入文獻之擷取資料

| Ethnicity | Author, Year | Case/control | Genotype Frequencies^a^ | | Allele distribution^b^ | | |
| --- | --- | --- | --- | --- | --- | --- | --- |
|  |  |  | Case | Control | Case | Control | |
| Caucasian | **Moos, 2000** | 55/240 | 36/18/1 | 166/74/0 | 90/20 | | 406/74 |
|  | **Sezgin**, 2008 | 151/84 | 121/26/4 | 72/12/0 | 268/34 | | 156/12 |
|  | Munoz-Valle, 2014 | 100/100 | 88/12/0 | 93/7/0 | 188/12 | | 193/7 |
|  | Vunkov, 2016 | 117/94 | 84/31/2 | 65/26/3 | 199/35 | | 156/32 |
|  | Rogoveanu, 2018 | 90/215 | 73/17/0 | 173/40/2 | 163/17 | | 386/44 |
|  | Sobhan, 2018 | 110/120 | 79/30/1 | 85/33/2 | 188/32 | | 203/37 |
|  | Fernandes, 2020 | 92/165 | 49/27/16 | 112/34/19 | 125/59 | | 258/72 |
| Egyptian | Abdel Galil, 2017 | 210/210 | 180/25/5 | 115/82/13 | 385/35 | | 312/108 |
|  | Raafat, 2020 | 90/90 | 51/37/2 | 76/8/6 | 139/41 | | 160/20 |
| Asian | Han, 2012 | 301/291 | 79/188/34 | 258/33/0 | 346/256 | | 549/33 |
|  | Ji, 2013 | 200/305 | 143/50/7 | 253/50/2 | 336/64 | | 556/54 |
|  | Chen, 2018 | 249/301 | 120/96/33 | 142/140/19 | 336/162 | | 424/178 |
|  | This study | 591/536 | 484/95/12 | 428/104/4 | 1063/119 | | 960/112 |

^a^ :the genotype frequencies are shown in the order of GG/AG/ AA; ^b^ :the allele distribution are shown in the order of G/A
