# Supplementary material for: Genetic association between TNF-α G-308A and osteoarthritis in Asians: A case–control study and meta-analysis
Source: PLoS One. 2021 Nov 4;16(11):e0259561. doi: 10.1371/journal.pone.0259561 (PMC8568107; doi:10.1371/journal.pone.0259561)
Supplement: S5 Table — (DOCX) [file pone.0259561.s005.docx]

| **S5 tab****le、TNF-α G-308A文獻品質評估** | | | | | | | | | |
| --- | --- | --- | --- | --- | --- | --- | --- | --- | --- |
|  | Selection | | | | Comparability | | Exposure | | |
| Author, year | Is the case definition adequate | Representativeness of the cases | Selection of Controls | Definition of Controls | Study controls for age | Study controls for additional factors | Ascertainment of exposure | Same method of ascertainment for cases and controls | Non-Response rate |
| Raafat, 2020 | a | a | c | a | a | a | a | a | b |
| Fernandes, 2019 | a | a | b | a | a | b | a | a | b |
| Chen, 2018 | a | a | b | a | a | b | a | a | b |
| Sabhan, 2018 | a | a | b | a | a | a | a | a | b |
| Rogoveanu, 2018 | a | a | b | a | a | b | a | a | b |
| Galil, 2017 | a | a | b | a | a | b | a | a | b |
| Vnulov, 2016 | a | a | c | a | a | a | a | a | b |
| Valle, 2014 | a | a | a | a | a | a | a | a | b |
| Ji, 2013 | a | a | a | a | a | a | a | a | b |
| Han, 2012 | a | b | c | a | a | b | a | a | b |
| Sezgin, 2008 | a | a | b | a | a | b | a | a | b |
| Moos, 2000 | a | a | b | a | a | b | a | a | b |
| **Is the case definition adequate?** a: yes, with independent validation b: yes, e.g. record linkage or based on self-reports; c: no description.  **Representativeness of the cases**: a: consecutive or obviously representative series of cases; b: potential for selection biases or not stated.  **Selection of Controls**: a: community controls; b: hospital controls; c: no description.  **Definition of Controls**: a: no history of disease (endpoint); b: no description of source.  **Study controls for age and sex**: a: yes; b: no.  **Study controls for over than two additional factors**: a:yes; b: no.  **Ascertainment of exposure**: a: secure record (e.g. surgical records); b: structured interview where blind to case/control status; c: interview not blinded to case/control status; d: written self-report or medical record only; e: no description.  **Same method of ascertainment for cases and controls**: a: yes; b: no.  **Non-Response rate**: a: same rate for both groups; b: no respondents described; c: rate different and no designation. | | | | | | | | | |
