# Supplementary figures and images for: Genetic association between TNF-α G-308A and osteoarthritis in Asians: A case–control study and meta-analysis
Source: PLoS One. 2021 Nov 4;16(11):e0259561. doi: 10.1371/journal.pone.0259561 (PMC8568107; doi:10.1371/journal.pone.0259561)

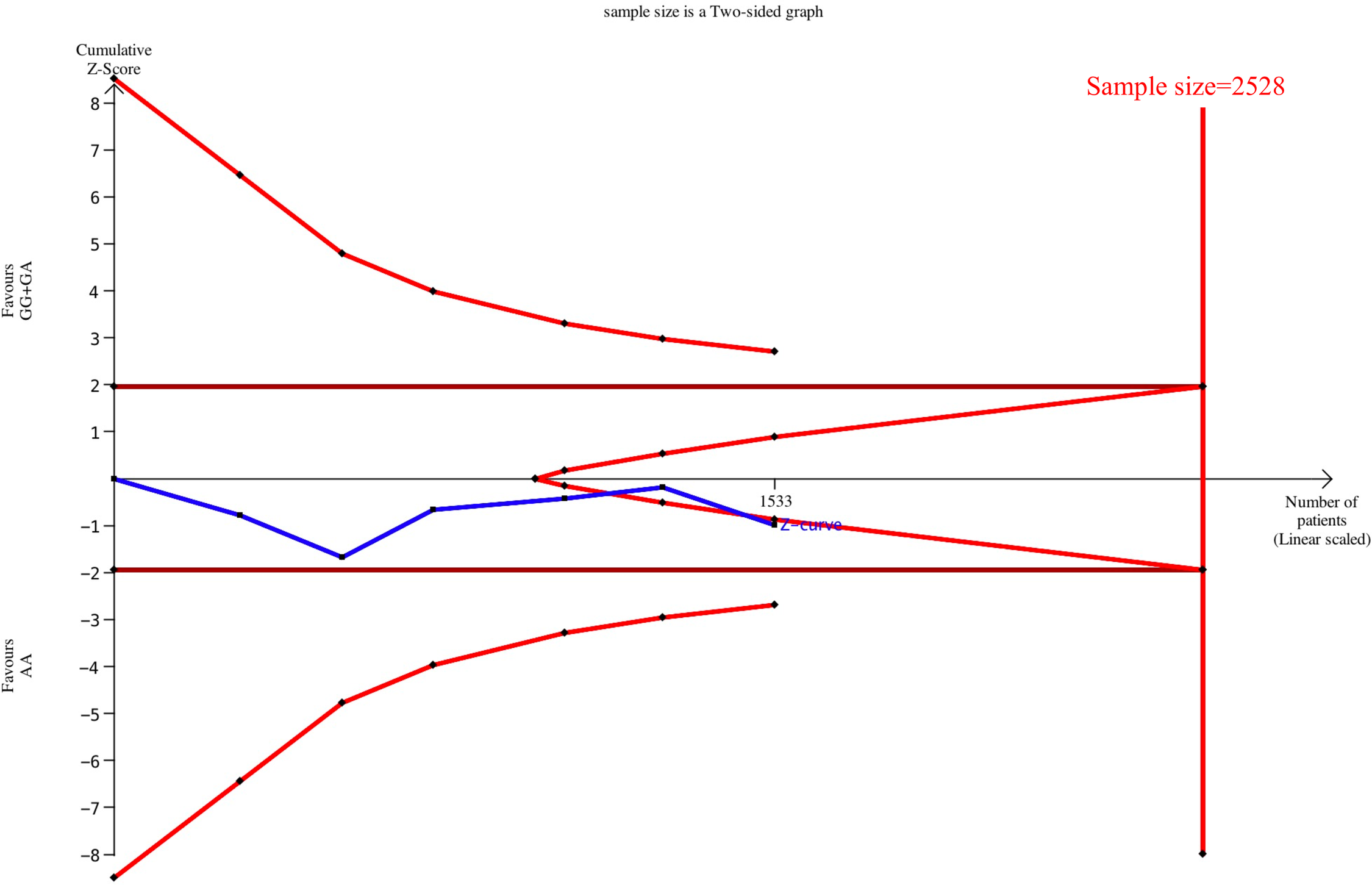

Supplement: S1 Fig — A trial sequential analysis was performed using a dominant model assumption. Detailed settings: significance level = 0.05, power = 0.8, least extreme odds ratio to be detected = 1.5, minor allele frequency = 0.069, and I2 (heterogeneity) = 0%. (TIF) [file pone.0259561.s006.tif]

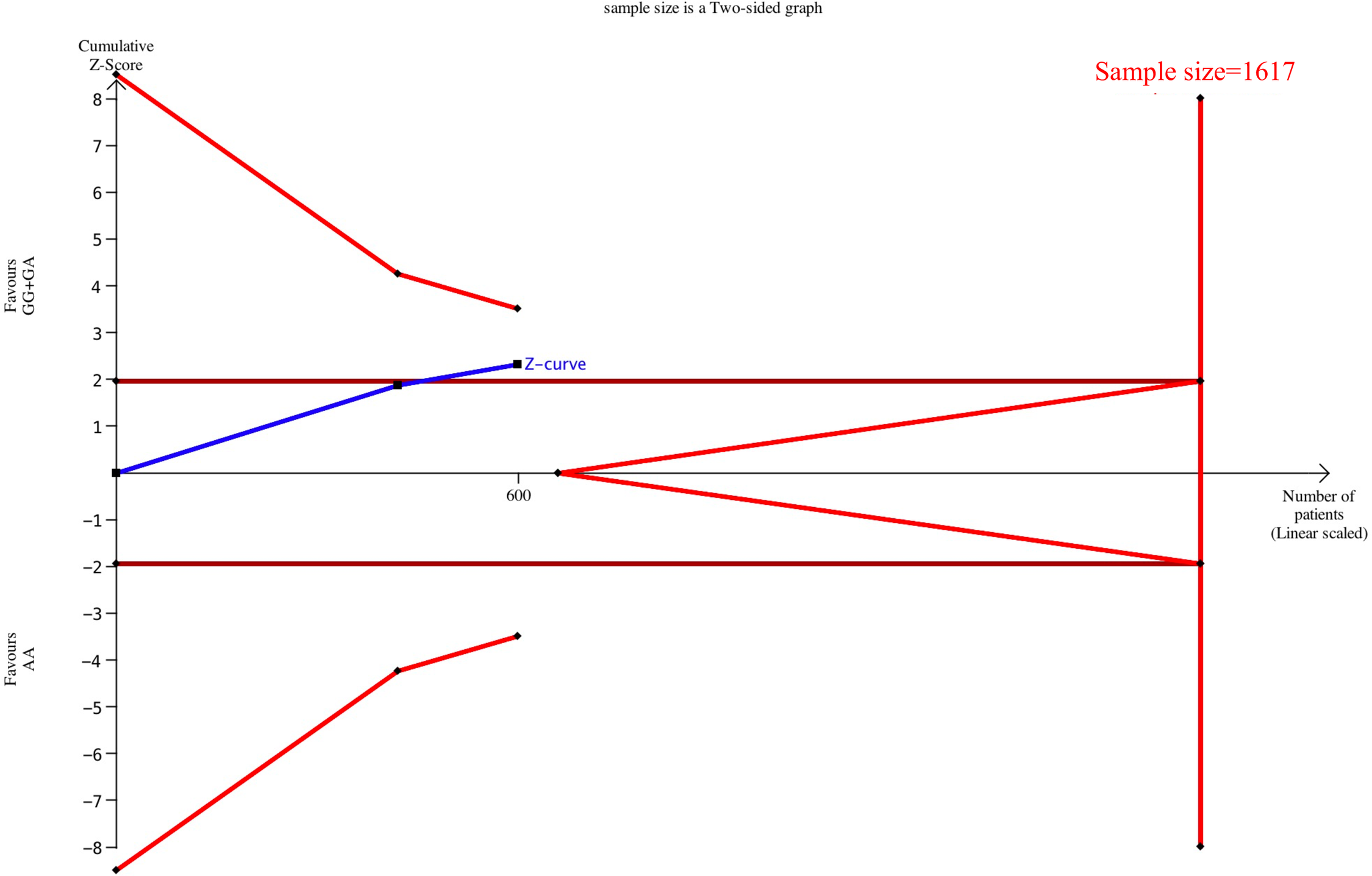

Supplement: S2 Fig — A trial sequential analysis was performed using a dominant model assumption. Detailed settings: significance level = 0.05, power = 0.8, least extreme odds ratio to be detected = 1.5, minor allele frequency = 0.12, and I2 (heterogeneity) = 0%. (TIF) [file pone.0259561.s007.tif]

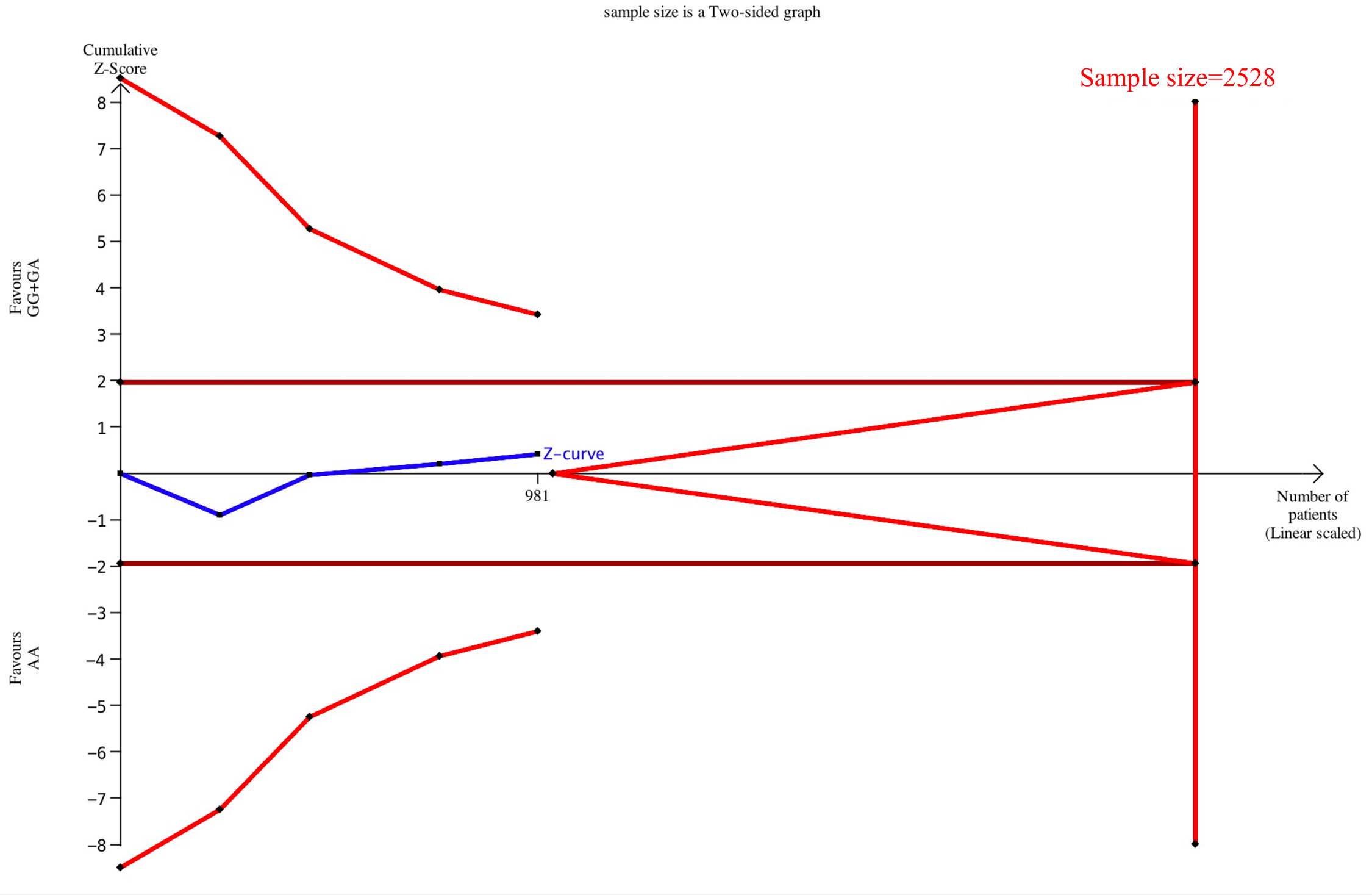

Supplement: S3 Fig — A trial sequential analysis was performed using a dominant model assumption. Detailed settings: significance level = 0.05. power = 0.8, least extreme odds ratio to be detected = 1.5, minor allele frequency = 0.069, and I2 (heterogeneity) = 0%. (TIF) [file pone.0259561.s008.tif]
